# Multiple macroevolutionary routes to becoming a biodiversity hotspot

**DOI:** 10.1126/sciadv.aau8067

**Published:** 2019-02-06

**Authors:** J. Igea, A. J. Tanentzap

**Affiliations:** Ecosystem and Global Change Group, Department of Plant Sciences, University of Cambridge, Downing St., Cambridge CB2 3EA, UK.

## Abstract

Why is species diversity so unevenly distributed across different regions on Earth? Regional differences in biodiversity may stem from differences in rates of speciation and dispersal and colonization times, but these hypotheses have rarely been tested simultaneously at a global scale. Our study reveals the macroevolutionary routes that have generated hotspots of mammal and bird biodiversity by analyzing the tempo and mode of diversification and dispersal within major biogeographic realms. Hotspots in tropical realms had higher rates of speciation, whereas those in temperate realms received more immigrant species from their surrounding regions. We also found that hotspots had higher spatial complexity and energy availability, providing a link between the environment and macroevolutionary history. Our study highlights how assessing differences in macroevolutionary history can help to explain why biodiversity varies so much worldwide.

## Teaser

Global hotspots of biodiversity arise from faster species generation in the tropics and higher migration in temperate realms.

## INTRODUCTION

Biodiversity is extremely unevenly distributed across the globe and understanding why has long fascinated biologists (*1*). For example, there are many exceptions to the tendency for species richness (SR) to increase toward the equator—widely studied as the latitudinal diversity gradient (*1*–*3*). This finer-scale association between biodiversity and geography (*4*) is exemplified by the 35 terrestrial biodiversity hotspots proposed by Myers *et al.* (*5*, *6*) for conservation purposes based on plant endemicity and habitat loss. A third of Myers’ hotspots were located in temperate zones and were more diverse than many regions closer to the equator, demonstrating that high levels of SR can also be found outside the tropics.

Regional differences in biodiversity may ultimately arise through at least one of three macroevolutionary routes. First, differences in historic rates of in situ diversification (i.e., speciation minus extinction) can result in more species accumulating in some areas than others. Second, differences in historic rates of lineage dispersal can result in some areas acting as sources of species that are exported elsewhere and some that are sinks that import species (*7*). Last, an older age of colonization of a region may promote diversity if there was more time to accumulate species, generating “museums” of biodiversity (*8*), as formalized in the “time-for-speciation” hypothesis (*9*, *10*). However, most studies of regional diversity patterns have not compared the relative importance of these different potential routes (*3*).

The three macroevolutionary routes giving rise to regional differences in biodiversity are at least partially paved by the environment (*1*). Some environmental variables may favor cladogenesis, such as past tectonic movements that generate isolation (*11*), while others may favor the establishment of immigrant species, such as historically stable climates that create regional refuges for species during periods of global change (*12*) and favor dispersal from environmentally similar areas, such as because of niche conservatism (*13*). Other environmental variables may favor both cladogenesis and immigration. For example, higher environmental energy might promote speciation by increasing mutation rates and shortening generation times (*14*) and also allow regions to hold more species by expanding their carrying capacity (*15*). Similarly, a higher speciation rate and local carrying capacity are both associated with physiographic heterogeneity (*16*, *17*) and habitat complexity (*18*, *19*).

Here, we use terrestrial hotspots of mammal and bird biodiversity to understand how different macroevolutionary routes (*20*) generate extreme spatial differences in species diversity. We delineated hotspots using the number of species in an area divided by the inverse of range of those species. Hotspots based on this measure—known as weighted endemicity (WE) (*21*) and not to be confused with counting endemic species—identify the contribution of each area to global biodiversity more accurately than SR, because widespread species are not counted in every area where they occur and so do not have a disproportionate influence on the metric. Therefore, hotspots based on WE will be more representative of the distribution of biodiversity across multiple regions on Earth than hotspots based on SR, which are exclusively centered in the tropics. Using diversification rate and historical biogeography inference methods, we then tested which macroevolutionary routes could better explain the existence of mammal and bird hotspots across different regions on Earth. Efforts to reconstruct explicitly the historical rates of migration and diversification of biodiversity hotspots have largely focused on small clades or specific geographic regions (*20*, *22*, *23*), without a broader global context.

## RESULTS AND DISCUSSION

We first used global species maps (*24*, *25*) to delineate hotspots. After overlaying species ranges with a grid of 100 km by 100 km cells, we defined separate mammal and bird hotspots for subsequent analyses as the richest 20% of cells in terms of WE (fig. S1, A and B). We chose this threshold to obtain clade-specific hotspots that were roughly equivalent in size to Myers’ hotspots (*5*, *6*). Despite being partially delineated using habitat threat, we used Myers *et al.* (*5*) as a basis for comparison because they identified large spatial unevenness in biodiversity. They observed that 20% of the global land area was sufficient to retain many of the most biodiverse biomes on the planet (e.g., Andes, Sundaland, Madagascar, and Mediterranean Basin) and >40% of all vertebrate species (*6*). Our resulting WE-based hotspots were broadly overlapped both the Myers’ hotspots and the hotspots based on total SR (see Materials and Methods), suggesting that all the measures captured a similar biological pattern.

**Fig. 1:**
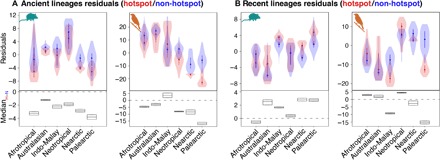
Hotspots are poor in ancient lineages and sometimes rich in recent lineages. Residuals from linear models predicting cell-specific richness of (**A**) ancient and (**B**) recent lineages in hotspots (H; shown in red) and non-hotspot regions (N; shown in blue). Positive residuals indicate a regional excess of ancient/recent lineages, and negative residuals indicate a deficit. The bottom panels show the median of the difference between a random hotspot point and a random non-hotspot point and the 95% confidence interval around that median calculated with a Wilcoxon rank sum test.

Using hotspots and neighboring regions within six biogeographic realms, we assessed the spatial variation in accumulation of ancient and recent lineages. We first obtained species-specific rates of diversification by estimating the diversification rate (DR) metric (*26*) for the most comprehensive phylogenies of mammals and birds (see Materials and Methods). DR captures the number of historic diversification events that lead to a given species, weighted by the relative age of those events, but does not explicitly model extinction (*26*, *27*). We then built linear regression models that predicted the richness of species that were either ancient [i.e., older, with DR values in the first quartile (Q1) of the distribution] or recent [younger, with DR values in the fourth quartile (Q4) of the distribution]. Total SR in each cell (*27*) was the sole model predictor. By examining the residuals of these linear models, we determined which cells had an excess or a deficit of species in each of the two quartiles within each biogeographic realm (*27*). Our results revealed that hotspots generally had a deficit of ancient lineages and an excess of recent lineages when compared to neighboring regions, except in birds where evidence of the latter was mixed (Fig. 1). We confirmed that these results were robust both to phylogenetic uncertainty and to the method used for estimating diversification rates (fig. S2, table S1, and see Materials and Methods).

**Fig. 2:**
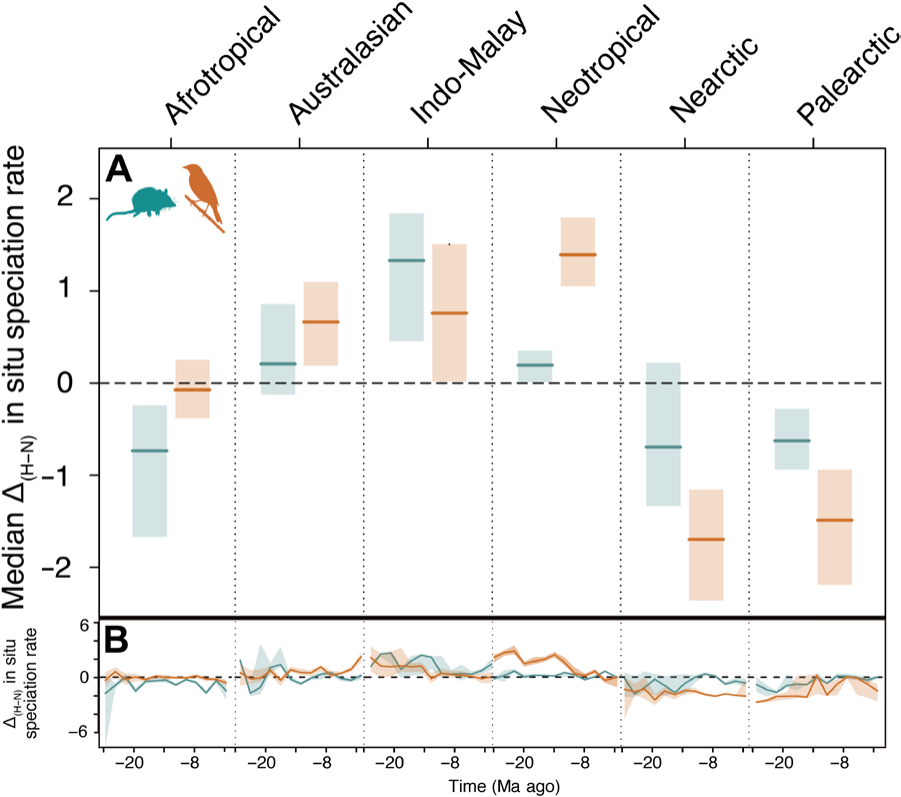
Contrasting rates of in situ cladogenesis in hotspots compared to surrounding non-hotspot regions. (**A**) In situ cladogenesis rates between 2 to 26 Ma ago within non-hotspots were subtracted from rates within hotspots in each of six biogeographic realms and divided by the overall SD to allow for comparison across realms. Solid lines indicate median differences (Δ) ± 90% confidence interval. Intervals overlapping the dotted line indicate a lack of statistically significant differences at α = 0.10. (**B**) Differences for each 2-Ma time bin.

**Fig. 3:**
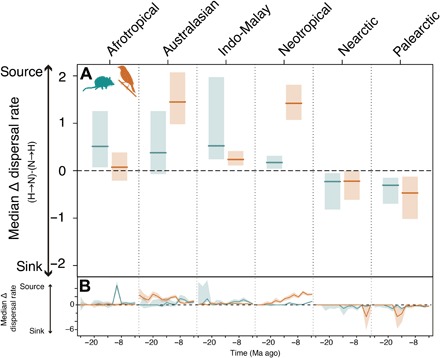
Source-sink dynamics of hotspots and their surrounding regions. (**A**) Dispersal rates between 2 and 26 Ma ago from non-hotspots to hotspots (N → H) were subtracted from hotspot to non-hotspots (H → N) rates within each realm. Lines and shaded areas presented as in Fig. 2.

Next, we assessed whether differences in macroevolutionary routes generated the different patterns of accumulation of ancient and recent species in hotspot and non-hotspot regions across biogeographic realms. We found that the general deficit of ancient lineages and more variable excess of recent lineages in the hotspots compared to nearby regions resulted from contrasting macroevolutionary histories across biogeographic realms. We reached this conclusion by reconstructing assembly dynamics within hotspots and non-hotspots of each realm using historical biogeographic inference (*28*). We found that both mammal and bird species were generated at faster rates in the past 25 million years (Ma) within hotspots compared with nearby regions of largely tropical realms such as Australasia, Indo-Malay, and Neotropics. By contrast, cladogenetic rates were similar or lower than surrounding areas in hotspots of the Afrotropics and temperate Palearctic and Nearctic realms (Fig. 2). Therefore, in situ cladogenesis could explain the accumulation of biodiversity in most tropical but not temperate hotspots. In temperate but not tropical realms, greater rates of historical dispersal rather than in situ cladogenesis could explain the accumulation of mammal and bird diversity within hotspots (Fig. 3). We found no evidence that colonization age alone could explain the differences in biodiversity, as hotspots were generally colonized later than non-hotspot regions in temperate realms, and there was no consistent difference in the age of colonization across tropical realms (fig. S3A).

Together, our findings suggest that contrasting macroevolutionary routes have shaped the uneven distribution of biodiversity across biogeographic realms. In all primarily tropical realms, except the Afrotropics, hotspots consistently generated and exported species at higher rates than their nearby areas, whereas the disproportionate richness in hotspots of temperate realms could be explained by greater rates of immigration from surrounding regions. The Afrotropics may lay somewhere outside these two routes. Afrotropical hotspots did not generate species more quickly or import them at greater rates in the past 25 Ma. The region became more arid during the late Miocene and early Pliocene as the Sahara Desert was formed (*29*). This change in the regional climate could have generated differences in the extinction dynamics of the Afrotropics hotspots compared to the non-hotspots. However, we could not estimate these extinction dynamics with the available methods. Hotspot diversity may have also been greater for ecological rather than evolutionary reasons, e.g., greater niche space (*15*).

Our results were generally robust to the methodological assumptions. First, SR is positively correlated with region size (*30*), but we found no evidence that the difference in size between hotspots and non-hotspot regions could alone explain our results. As hotspots of endemicity were defined globally, there were large differences between the sizes of the hotspots and non-hotspot regions within each realm (table S2). To assess whether these differences could generate the different macroevolutionary patterns that we observed, we repeated our analysis by randomly sampling combinations of cells with similar size and spatial structure to the hotspot cells in each realm. We consistently found that the real estimates of in situ cladogenesis (fig. S4), dispersal (fig. S5), and colonization time (fig. S3B) lay outside the simulated distributions. Therefore, the differences in macroevolutionary patterns that we observed between hotspots and their surrounding areas must have stemmed from differences in species composition and/or environmental features rather than simply due to size. Second, we also found that hotspots were more clustered in space than non-hotspot cells across all realms, particularly in tropical as compared with temperate realms. However, the differences across realms were small and <10% in the most extreme case (table S3 and fig. S6). The slightly greater clustering of tropical hotspots is therefore unlikely to explain fully the different macroevolutionary routes that we found in tropical and temperate realms (fig. S6). Third, we confirmed that two alternative ways of delineating biodiversity hotspots were congruent with the results for the WE-based hotspots (see Supplementary Text and figs. S7 and S8). These alternate definitions used SR (fig. S1, C and D) and areas where narrow-ranged species (NRS) occurred (fig. S1, E and F), which have been proposed to reflect past opportunities for speciation (*31*).

**Fig. 4:**
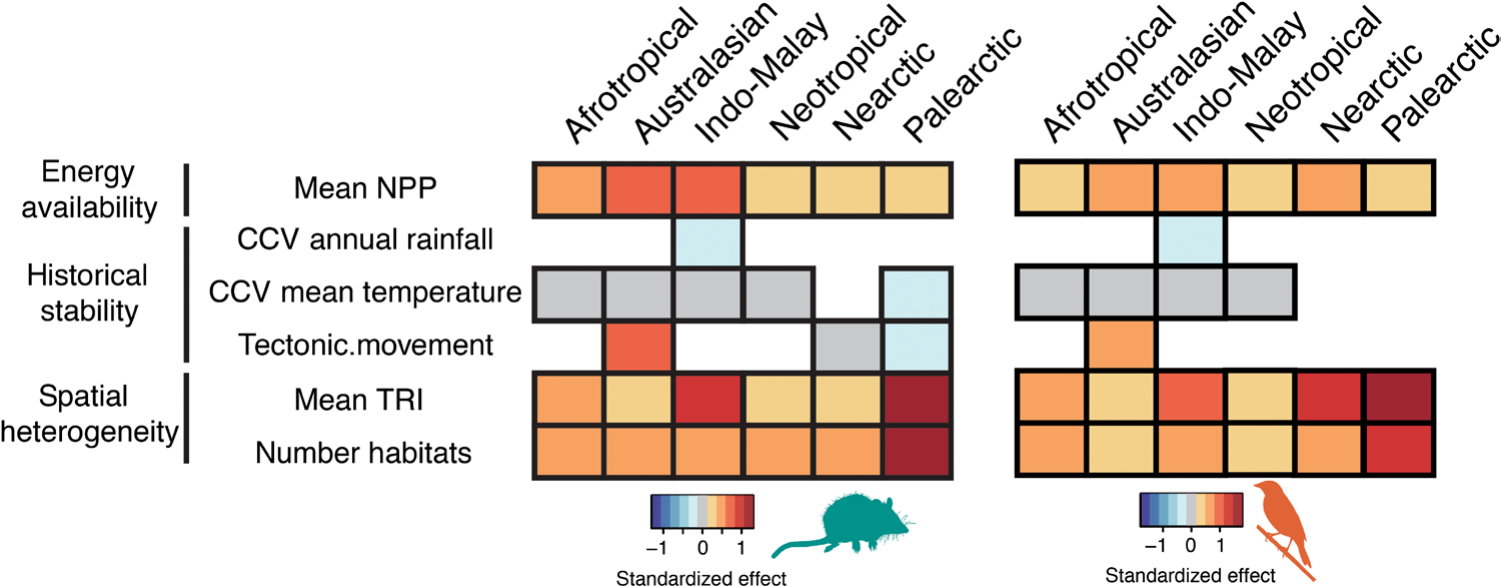
Hotspots are more spatially complex and have more energy than surrounding regions. Standardized differences in the average cell values of environmental variables between hotspots and non-hotspot cells within each of six biogeographic realms based on the coefficients of univariate spatially autocorrelated linear regressions. Blank areas indicate nonsignificant differences. CCV, climate change velocity.

Last, we found evidence that unique environments inside the hotspots could have promoted differences in macroevolution when compared to neighboring non-hotspot regions. Linear models with spatial autocorrelation allowed us to compare environmental features potentially associated with differences in rates of in situ diversification and dispersal between hotspots and their surrounding areas within each realm. We found that hotspots had a greater mean net primary productivity (NPP), terrain ruggedness index (TRI), and more habitats than their surrounding regions (Fig. 4). These differences were consistent across realms despite the contrasting macroevolutionary routes between tropical and temperate regions. This finding is not entirely unexpected because the same environmental features can be associated with contrasting macroevolutionary routes. Specifically, increased historic opportunities for speciation may have resulted from higher energy availability (*14*) and spatial complexity (*17*, *18*) in the tropics. In temperate regions, the same variables may have elevated carrying capacities and packed more immigrant species into the hotspots, which could have also acted as biodiversity refuges during past climate change (*32*, *33*). The velocity of late Quaternary climate change was not, however, different between hotspot and non-hotspot areas (Fig. 4), despite previously being shown to correlate negatively with vertebrate endemism (*12*). The extent of the tropics was also larger over much of the past 25 Ma (*3*). Thus, more immigrants could have dispersed into temperate latitudes, i.e., “out-of-tropics” hypothesis (*34*), providing an additional explanation for the different macroevolutionary routes that we observed across realms.

Our study offers an integrative approach to understanding why biodiversity varies so much across the globe using a regional-scale and spatially explicit reconstruction of historical dispersal and diversification alongside an analysis of ecological gradients. In general, vertebrate, invertebrate, and plant diversity are spatially correlated at regional scales across the planet (*4*), so we expect similar mechanisms to generate biodiversity across the Tree of Life (*35*). Similar analyses carried out in other groups may nevertheless result in clade-specific idiosyncrasies. For instance, the relative roles of in situ cladogenesis and dispersal as drivers of regional diversity may be different in taxa with lower vagility than mammals and birds, such as amphibians and insects (*36*, *37*). By simultaneously comparing different macroevolutionary routes and their macroecological features in two major vertebrate clades, our study now provides a new answer to the old question of why diversity varies so much across the world.

## MATERIALS AND METHODS

### Phylogenetic and species distribution data

The maximum clade credibility (MCC) tree for mammals was estimated with 100 random trees from the pseudoposterior provided by Kuhn *et al.* (*38*) using TreeAnnotator v.1.8.2 (*39*). Following Rolland *et al.* (*40*), we recalibrated the dates in this MCC tree and in the initial 100 trees from the pseudoposterior with alternative dates from Meredith *et al.* (*41*) using PATHd8 (*42*). For birds, we used 100 random trees from the updated version of the posterior distribution of trees in Jetz *et al.* (*26*), and we obtained the MCC tree as above.

Species distribution data were obtained from the International Union for Conservation of Nature (IUCN) Red List for mammals (version 5.2) (*24*) and BirdLife (version 6.0) (*25*) for birds. Marine species were not analyzed. Hotspots were defined using all species with distribution data—5302 and 11,093 species of mammals and birds, respectively. Species names in the phylogenetic trees were standardized with the IUCN (version 5.2) and BirdLife (version 6.0) taxonomies and collated with the distributional data. In total, 4633 and 9622 species of mammals and birds, respectively, representing 83.3 and 86.5% of the described species were present in the phylogenetic trees and were used in downstream analyses. We followed the definition of the World Wildlife Fund Simplified Biogeographical Realms (version 2.0) (*43*) for the different biogeographic realms. By definition, the areas in each realm share a common evolutionary and biogeographic history, so comparing them provides a framework for generalization across realms with distinct biotas (*43*). The Oceanic realm was much smaller than the rest, so we did not include it in our analyses.

### Definition of the biodiversity hotspots

We overlaid the distributions of mammals and breeding ranges of birds onto a 100 km by 100 km grid to define hotspots of biodiversity (fig. S1, A and B). WE in each grid cell was calculated by weighting each species’ occurrence by the inverse of its corresponding range and then summing values across all species in a cell. We then defined the hotspots as the cells with the top 20% of WE values. This definition covered 19.9% of our grid surface, roughly equivalent in size to Myers’ hotspots that comprised 17.0% of global land surface (*6*), and resulted in 3826 and 3304 cells for mammals and birds, respectively. There was a 74.4% overlap between the mammal and bird hotspots and a 69.7 and 71.3% overlap between the Myers’ hotspots and the ones we defined for mammals and birds, respectively (fig. S1). We also found an overlap between our WE-based hotspots and hotspots defined simply with total SR of 56.9 and 62.4% for mammals and birds, respectively. Overlap was expected since WE and SR were positively correlated (Spearman’s ρ = 0.74 in our dataset).

We also assessed whether alternative ways of delineating biodiversity hotspots changed our results. First, we defined hotspots based on SR. Hotspots were defined as the cells with the top 20% values of SR and, as expected, were concentrated in the tropics (fig. S1, C and D). Two realms, Australasia and Nearctic, had very few or no hotspot cells or showed a very small overlap with the WE hotspots (table S4). Therefore, we were only able to carry out further analyses within the Afrotropics, Indo-Malay, Neotropics, and Palearctic. Second, we delineated alternative hotspots as “centers of endemism” where NRS were found. These regions have been proposed to provide many opportunities for past speciation while enabling the survival of narrow-ranged endemics due to stable environments (*31*). Following Jetz *et al.* (*31*), we defined NRS as species with a range of ≤100,000 km^2^ (10 cells in our grid) and hotspots as all the cells where these species were found (*n*_mammals_ = 2723 cells, *n*_birds_ = 2041 cells). The resulting hotspots substantially overlapped with the WE-based hotspots, particularly for mammals (fig. S1, E and F, and table S4). We repeated the diversification rate and historical biogeography analyses described in the Results and Discussion for the SR- and NRS-based hotspots and found that the results were largely congruent with the WE-based hotspots (see Supplementary Text and figs. S7 and S8).

### Species diversification rates

The DR metric (*26*) was calculated as the number of nodes that separated each species from the root of the tree weighted by the distance of each node to the present. Thus, DR represents the relative phylogenetic isolation of species and has been used to determine regions that are enriched in actively diversifying lineages and older lineages (*27*, *44*). We divided the species into four quartiles, with older lineages (here termed “ancient”) in Q1 and younger, actively diversifying lineages (“recent”) in Q4. Using simple linear regression models, we predicted Q1 and Q4 SR from the total SR in each cell. Positive residuals of the corresponding linear model indicated that a particular cell contained an excess of ancient or recent lineages, respectively, while negative residuals indicated a deficit (*27*, *44*). For each biogeographic realm, we then assessed whether an average hotspot cell had different values for the Q1 and Q4 residuals when compared to an average non-hotspot cell. To do this, we ran two spatial simultaneous autoregressive (SAR) error models, each with the Q1 and Q4 residuals as a response and hotspot category as a predictor using the R package spdep (*45*). Last, we compared the mean values of Q1 and Q4 SAR residuals in hotspots and non-hotspot cells of each biogeographic realm using a Wilcoxon rank sum test.

We also analyzed the effect of phylogenetic uncertainty in our estimates of DRs. We obtained DR estimates for 100 random trees from the pseudoposterior distribution of the mammal and bird phylogenies (see above). We then verified that the MCC-based DR estimates were positively correlated with the median DR values across the 100 trees (fig. S2A) and with each individual tree (fig. S2B). We also verified that the residuals of the DR quartile linear regressions with the MCC tree were correlated with the residual values across the 100 trees (fig. S2, C and D).

DR has been shown to correlate to recent speciation events. However, it does not account for extinction and temporal variation in diversification rates within lineages (*26*, *27*). To check whether these assumptions could influence our analyses, we estimated species-specific rates of speciation and extinction (i.e., “tip rates”) in the mammal and bird phylogenies using Bayesian analysis of macroevolutionary mixtures (BAMM) (*46*). BAMM models diversification rate heterogeneity across lineages and through time. Although the reliability of BAMM has recently been questioned, a focus of criticism was the influence of unobserved rate shifts within extinct lineages on diversification estimates (*47*). This issue affects most methods, but the contribution of shifts in extinct lineages to the overall probability of extinction is marginal under biologically plausible scenarios (*48*). A related concern is that the extinction rate estimates obtained from molecular phylogenies of extant species may sometimes be unreliable (*49*, *50*). Accurate speciation rates and relative diversification rates can, however, be routinely inferred using a variety of methods (*51*), including BAMM. Simulation studies have shown that BAMM infers speciation rates with high accuracy, particularly for large, well-sampled trees such as ours (*48*), and can robustly estimate speciation rates in the absence of paleontological data (*52*). Despite having different assumptions, BAMM and DR estimates of diversification are generally positively correlated (*53*), including in our dataset (fig. S3, E and F; Pearson’s *r*_mammals_ = 0.63, *r*_birds_ = 0.58; for both, *P* < 0.0001). This result suggests that both measure capture similar information about diversification. We also found that residuals of DR- and BAMM-based quartile regressions were positively correlated (fig. S3, E and F; Pearson’s *r*_mammals.Q1_ = 0.27, *r*_mammals.Q4_ = 0.68, *r*_birds.Q1_ = 0.65, *r*_birds.Q4_ = 0.77; for all, *P* < 0.0001). Similarly, comparisons between hotspots and non-hotspot regions across realms using DR residuals (Fig. 1) largely matched those obtained using BAMM residuals (table S1).

### Historical biogeography analysis

We inferred the historical biogeography of mammals and birds using the R package Biogeography with Bayesian (and Likelihood) Evolutionary Analysis in R Scripts (BioGeoBEARS) (*28*, *54*). BioGeoBEARS infers biogeographic history using phylogenetic trees and locality data. Ancestral ranges were estimated by implementing a series of biogeographic models in BioGeoBEARS that allowed for different types of range shifts and that could be compared in a common likelihood framework. Here, we used the “small-sample” corrected Akaike Information Criterion (AICc) to compare the fit of six biogeographical models: dispersal-extinction-cladogenesis (DEC) (*55*), dispersal-vicariance analysis (DIVA) (*56*), Bayesian analysis of biogeography (BAYAREALIKE) (*57*), and versions of the previous three models that allowed for founder-event speciation (+J) (*54*), i.e., DEC+J, DIVA+J, and BAYAREALIKE+J. All the models account for anagenetic dispersal and local extinction and differ in the cladogenetic events that they allow: DEC models subset sympatry and narrow vicariance, DIVA models subset narrow and widespread vicariance, and BAYAREA models subset narrow and widespread sympatry [see (*28*) for details]. To alleviate computational issues related to an excessive number of states (i.e., geographic areas), we performed six independent analyses, one for each biogeographic realm. In each analysis, we used the complete phylogenetic tree and coded the species as present in a maximum of two areas: hotspots in a focal realm, non-hotspot areas in a focal realm, and in any one of the remaining five realms. No constraints to dispersal were set between areas. Presence was assigned when at least 20% of the total species range overlapped a focal region. We then used the parameter rate estimates of the best fitting model (table S5) for each realm to perform 50 biogeographic stochastic mappings (BSMs) (*58*).

The BSMs first allowed us to count, date, and extract the chronology of anagenetic and cladogenetic events in a probabilistic sample of biogeographic histories for hotspots and non-hotspot regions within each realm. We then binned the events into 2-Ma periods and calculated rolling estimates of the rates of regional dispersal out of and into each region following Xing and Ree (*23*). Dispersal rates for each region were calculated as the median number of dispersal events in a time bin across the BSMs divided by the number of lineages present in that region in the previous time bin. For instance, dispersal rates at 2 to 4 Ma from non-hotspots into hotspots (N → H) in the Afrotropics were calculated as the median number of N → H events at 2 to 4 Ma divided by the median number of lineages estimated in the hotspots in the Afrotropics at 4 to 6 Ma. To calculate rates of cladogenesis, we first used the BSMs to assign nodes to particular geographic regions. We then obtained the branch estimates of speciation rates for the complete phylogenetic trees with BAMM using the R package BAMMtools (*59*). Last, we calculated the rate of cladogenesis for a given region (hotspot or non-hotspot) in a time bin as the average of the speciation rates of the branches assigned to that region in that time bin. We discarded estimates that were older than 26 Ma, due to the lack of data (i.e., ≤10 lineages in each region), and younger than 2 Ma, to avoid confounding effects of ongoing speciation events (*26*).

### Size effect in historical biogeography analysis

We assessed whether the differences in size between hotspot and non-hotspot regions within realms could explain the differences in macroevolutionary patterns that we observed. We first fitted a cluster point process model to the point pattern of the hotspots in each realm using the R package spatstat (*60*). We used these models to simulate 50 sets of “control” hotspots with similar size and spatial structure to the real datasets (fig. S9). Last, we repeated the biogeographic inference and the BSMs as detailed above for each of the 50 control hotspots in each realm. We then compared whether the rate estimates obtained with the real datasets were significantly different from the distribution of rate estimates obtained with the simulated hotspots. We found differences between the real and the simulated datasets that suggested that size alone could not explain our results: (i) rates of in situ cladogenesis in the Palearctic and Nearctic hotspots were higher in the real than in the simulated datasets (fig. S4A); (ii) rates of in situ cladogenesis in the non-hotspot regions in the Australasia, Indo-Malay, and Neotropics were smaller in the real than in the simulated datasets (fig. S4B); and (iii) rates of dispersal from the hotspots into non-hotspot regions were consistently higher in the real than in the simulated datasets across all realms (fig. S5A).

### Contiguity of hotspots across biogeographic realms

We determined whether different spatial clustering of hotspot and non-hotspot areas within each realm could influence our results. For each realm, we calculated the median distance of each cell to all neighboring cells of the same class (hotspot or non-hotspot) with a radius of 1000 km (fig. S6). We then compared the differences of the mean distances among hotspots and non-hotspots using a *t* test.

### Environmental differences between hotspots and non-hotspots

We investigated whether there were significant differences between hotspots and surrounding non-hotspot regions for variables that have previously been implicated in generating biodiversity gradients (*12*, *14*–*19*). The models compared the mean values between hotspots and non-hotspots in the same realm for variables related to the following: (i) current energy availability (NPP), (ii) spatial heterogeneity (number of habitats and TRI), and (iii) historical stability (climate change velocity since the Last Glacial Maximum for mean annual rainfall and mean annual temperature and past tectonic movements). We used SAR error models that controlled for spatial autocorrelation as implemented in the R package spdep (*45*), with hotspot category as a predictor and each environmental variable as a response. The spatial neighborhood matrix was calculated for neighbors within 1000 km. Variables were centered and scaled to a mean of 0 and an SD of 1 before model fitting so as to generate standardized effects that would be comparable across the different variables. *P* values were Bonferroni-corrected for multiple comparisons. Data for NPP were obtained from the Socioeconomic Data and Applications Center (*61*). Climate change velocity was calculated following Sandel *et al.* (*12*), and climatic variables for the present and the Last Glacial Maximum (with the MIROC model) were obtained from WorldClim version 1.4 (*62*). We obtained habitat data from the U.S. Geological Survey (USGS) 1-km Global Land Cover Characteristics Data Base version 2.0 and used the Global 30 Arc-Second Digital Elevation Model (GTOPO30, also available from the USGS) to calculate cell-average values of TRI using the raster R package (*63*). Tectonic movement was calculated as the SD in the distances between a cell and its neighbors from 65 Ma until the present (*11*), as reconstructed with gplates (*64*). We also considered additional variables (mean elevation and elevation range), but they were highly correlated (Spearman’s ρ > 0.70) to other variables in our dataset and so removed before the analyses.

## Supplementary Material

http://advances.sciencemag.org/cgi/content/full/5/2/eaau8067/DC1

## SUPPLEMENTARY MATERIALS

Supplementary material for this article is available at http://advances.sciencemag.org/cgi/content/full/5/2/eaau8067/DC1 Supplementary Text Fig. S1. Global maps of the mammal and bird hotspots in this study (shown in red). Fig. S2. DR estimates are correlated across the pseudoposterior distribution and also correlate with BAMM estimates. Fig. S3. Age of colonization in hotspots and non-hotspots. Fig. S4. Empirically estimated in situ cladogenetic rates in hotspots and non-hotspots differ from rates estimated in “control areas” with similar size and spatial structure to the real hotspots. Fig. S5. Empirically estimated dispersal rates from hotspots to non-hotspots (H → N) and from non-hotspots to hotspots (N → H) differ from rates estimated in control areas with similar size and spatial structure to the real hotspots. Fig. S6. Similar differences in contiguity of hotspot and non-hotspot cells across biogeographic realms. Fig. S7. Species richness-based hotspots and narrow ranged species-based hotspots are poor in ancient lineages and sometimes rich in recent lineages. Fig. S8. Contrasting macroevolutionary routes in species richness-based hotspots and non-hotspots and in narrow ranged species-based hotspots and non-hotspots. Fig. S9. Example of simulating control hotspots. Table S1. DR and BAMM produce consistent differences between hotspot and non-hotspot regions. Table S2. Total size and proportion of hotspot cells across biogeographic realms. Table S3. Mean of median distances (kilometer) of each cell to every neighboring cell of the same class with a radius of 1000 km is shown for hotspots and non-hotspots for mammals and birds. Table S4. Overlap of WE-based hotspots with SR- and NRS-based hotspots. Table S5. Model fit of BioGeoBEARS in six biogeographic realms.
