# Supplementary material for: Multiple macroevolutionary routes to becoming a biodiversity hotspot
Source: Sci Adv. 2019 Feb 6;5(2):eaau8067. doi: 10.1126/sciadv.aau8067 (PMC6365113; doi:10.1126/sciadv.aau8067)
Supplement: http://advances.sciencemag.org/cgi/content/full/5/2/eaau8067/DC1 [file supp_5_2_eaau8067__index.html]

Science Advances | Science Advances

## Supplementary Materials

**This PDF file includes:**

- Supplementary Text
- Fig. S1. Global maps of the mammal and bird hotspots in this study (shown in red).
- Fig. S2. DR estimates are correlated across the pseudoposterior distribution and also correlate with BAMM estimates.
- Fig. S3. Age of colonization in hotspots and non-hotspots.
- Fig. S4. Empirically estimated in situ cladogenetic rates in hotspots and non-hotspots differ from rates estimated in “control areas” with similar size and spatial structure to the real hotspots.
- Fig. S5. Empirically estimated dispersal rates from hotspots to non-hotspots (H → N) and from non-hotspots to hotspots (N → H) differ from rates estimated in control areas with similar size and spatial structure to the real hotspots.
- Fig. S6. Similar differences in contiguity of hotspot and non-hotspot cells across biogeographic realms.
- Fig. S7. Species richness-based hotspots and narrow ranged species-based hotspots are poor in ancient lineages and sometimes rich in recent lineages.
- Fig. S8. Contrasting macroevolutionary routes in species richness-based hotspots and non-hotspots and in narrow ranged species-based hotspots and non-hotspots.
- Fig. S9. Example of simulating control hotspots.
- Table S1. DR and BAMM produce consistent differences between hotspot and non-hotspot regions.
- Table S2. Total size and proportion of hotspot cells across biogeographic realms.
- Table S3. Mean of median distances (kilometer) of each cell to every neighboring cell of the same class with a radius of 1000 km is shown for hotspots and non-hotspots for mammals and birds.
- Table S4. Overlap of WE-based hotspots with SR- and NRS-based hotspots.
- Table S5. Model fit of BioGeoBEARS in six biogeographic realms.

Download PDF

**Files in this Data Supplement:**

- Adobe PDF - aau8067\_SM.pdf
