# Supplementary material for: Multiple macroevolutionary routes to becoming a biodiversity hotspot
Source: Sci Adv. 2019 Feb 6;5(2):eaau8067. doi: 10.1126/sciadv.aau8067 (PMC6365113; doi:10.1126/sciadv.aau8067)
Supplement: http://advances.sciencemag.org/cgi/content/full/5/2/eaau8067/DC1 [file aau8067_SM.pdf]

Science Advances
AAAS

advances.sciencemag.org/cgi/content/full/5/2/eaau8067/DC1

# Supplementary Materials for

## Multiple macroevolutionary routes to becoming a biodiversity hotspot

J. Igea* and A. J. Tanentzap*

*Corresponding author. Email: ji247@cam.ac.uk (J.I.); ajt65@cam.ac.uk (A.J.T.)

Published 6 February 2019, *Sci. Adv.* **5**, eaau8067 (2019)
DOI: 10.1126/sciadv.aau8067

**This PDF file includes:**

Supplementary Text
Fig. S1. Global maps of the mammal and bird hotspots in this study (shown in red).
Fig. S2. DR estimates are correlated across the pseudoposterior distribution and also correlate with BAMM estimates.
Fig. S3. Age of colonization in hotspots and non-hotspots.
Fig. S4. Empirically estimated in situ cladogenetic rates in hotspots and non-hotspots differ from rates estimated in "control areas" with similar size and spatial structure to the real hotspots.
Fig. S5. Empirically estimated dispersal rates from hotspots to non-hotspots (H → N) and from non-hotspots to hotspots (N → H) differ from rates estimated in control areas with similar size and spatial structure to the real hotspots.
Fig. S6. Similar differences in contiguity of hotspot and non-hotspot cells across biogeographic realms.
Fig. S7. Species richness-based hotspots and narrow ranged species-based hotspots are poor in ancient lineages and sometimes rich in recent lineages.
Fig. S8. Contrasting macroevolutionary routes in species richness-based hotspots and non-hotspots and in narrow ranged species-based hotspots and non-hotspots.
Fig. S9. Example of simulating control hotspots.
Table S1. DR and BAMM produce consistent differences between hotspot and non-hotspot regions.
Table S2. Total size and proportion of hotspot cells across biogeographic realms.
Table S3. Mean of median distances (kilometer) of each cell to every neighboring cell of the same class with a radius of 1000 km is shown for hotspots and non-hotspots for mammals and birds.
Table S4. Overlap of WE-based hotspots with SR- and NRS-based hotspots.
Table S5. Model fit of BioGeoBEARS in six biogeographic realms.

## Supplementary Text

We first repeated the DR residuals and the historical biogeographic analyses described in the main text using the SR hotspots (fig. S15). We found largely congruent results with the WE-based hotspots: *i*) SR-based hotspots were generally poor in ancient lineages and rich in recent lineages, like WE-based hotspots (Fig. 1, fig. S11); *ii*) speciation was generally higher in hotspots than in non-hotspots in tropical realms, while the reverse was true in the Palearctic (Fig. 2, fig. S12a); and *iii*) hotspots imported species from non-hotspots at faster rates than they exported them in the Palearctic, and the reverse was true in tropical realms (Fig. 3, fig. S12b).

We similarly repeated the analyses using NRS-based hotspots (fig. S13). Similar to the main analyses based on WE, NRS-based hotspots were poor in ancient lineages and generally rich in recent lineages (Fig. 2, fig. S13). As in our main analysis, speciation was higher in non-hotspots than in hotspots in temperate realms. Similarly, in largely tropical realms, most of the parameters had the same sign as in our main analysis (i.e., speciation was generally larger in hotspots than in non-hotspots), but, in several instances, the intervals around the mean differences of speciation in hotspots minus non-hotspots overlapped with zero (Fig. 2, fig. S14a). Likewise, dispersal results for temperate realms matched the WE-based results but the differences in tropical realms were largely not statistically significant (Fig. 3, fig. S14b).

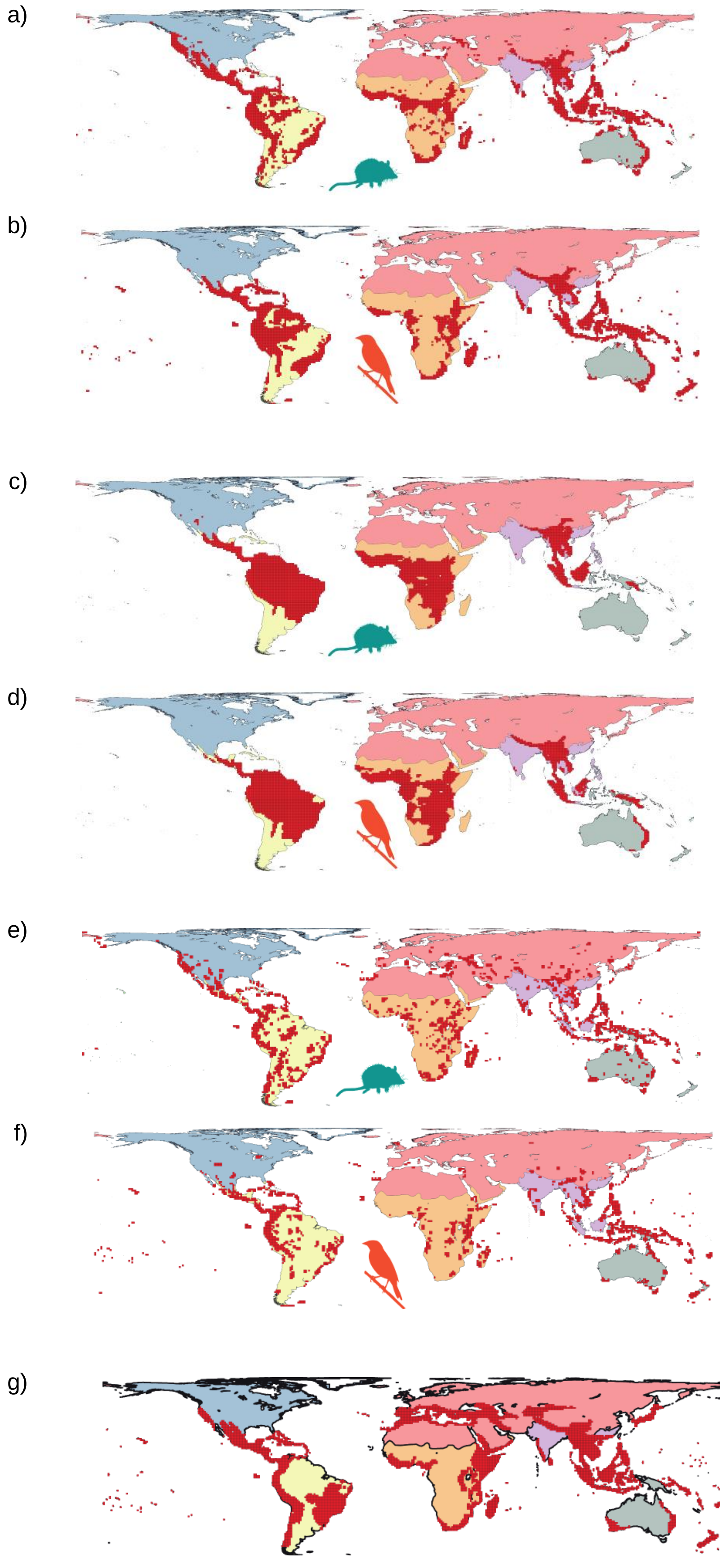
a)
b)
c)
d)
e)
f)
g)

**Fig. S1. Global maps of the mammal and bird hotspots in this study (shown in red).**

Hotspots delineated using weighted endemicity **(a**,**b)**; alternate hotspots using species richness (**c**,**d**); alternate hotspots using narrow-ranged species (**e**,**f**); and Myers conservation hotspots (**g**). Afrotropics are shown in orange, Australasia in grey, Indo-Malay in purple, Nearctic in blue, Neotropics in yellow and Palearctic in pink.

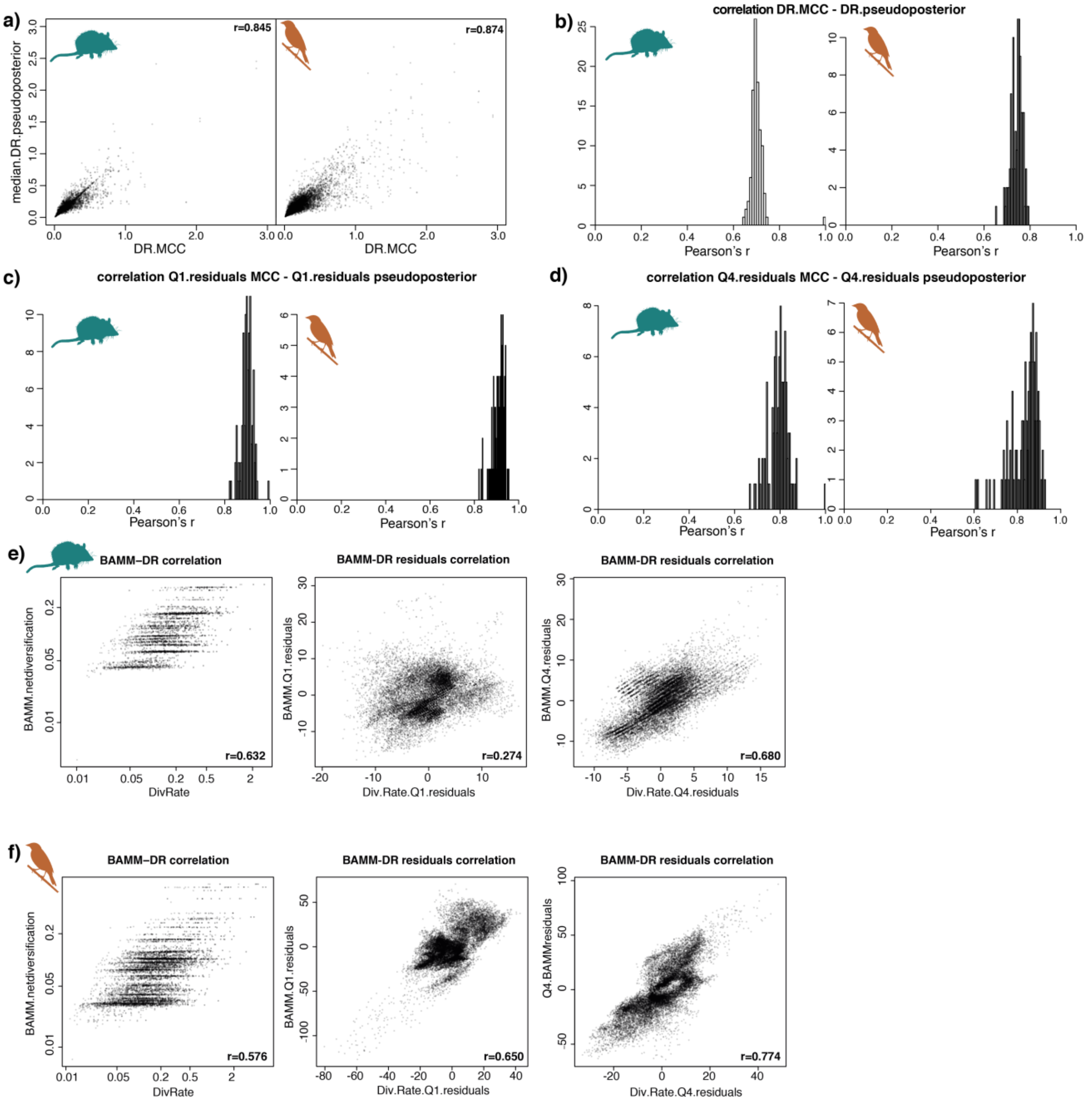


**Fig. S2. DR estimates are correlated across the pseudoposterior distribution and also correlate with BAMM estimates. a**) correlation of DR values from the MCC tree with the median values of DR across the 100 random trees from the pseudoposterior distribution; **b**) correlations of the DR values from the MCC with each of the 100 random trees from the pseudoposterior; **c**) correlations of the cell-specific ancient lineages residuals in the MCC and the 100 random trees; **d**) correlations of the cell-specific recent lineages residuals in the MCC and

the 100 random trees; **e**) (left to right) correlation of the species-specific DR values in the MCC tree and the species-specific values of net diversification calculated with BAMM; correlation of the cell-specific ancient lineages residuals calculated with DR and with BAMM; correlation of the cell-specific recent lineages residuals calculated with DR and with BAMM in mammals; and **f**) in birds.

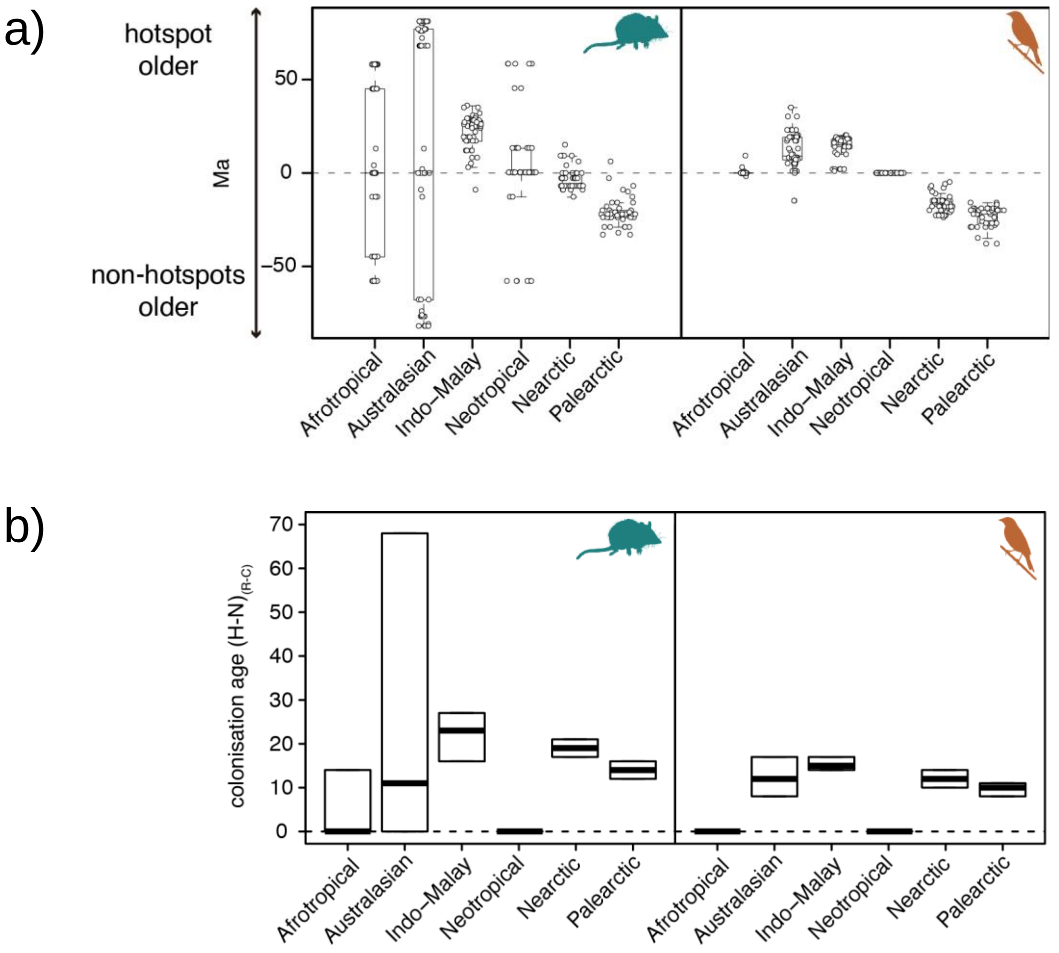


**Fig. S3. Age of colonization in hotspots and non-hotspots. a**) No consistent differences in colonization age between hotspot (H) and non-hotspot (N) regions across realms. Points represent each of the 50 biogeographic stochastic mappings (BSMs) in each realm. Colonization ages in the 50 BSMs were calculated as the first presence of a lineage in a region. Box limits indicate the first and third quartiles and whiskers indicate 1.5 times the interquartile range above the third quartile and below the first quartile. Differences overlapped zero (shown by dashed line) for most tropical realms, whereas non-hotspots were generally older later than non-hotspot regions, contrary to the prediction that greater diversity can arise from an older colonization date. **b**) Empirically-estimated differences in the age of colonization of hotspots and non-hotspots differ from rates estimated in “control areas” with similar size and spatial structure to the real

hotspots. Differences in the age of colonization between hotspots and non-hotspots (H – N) calculated with the real dataset (R) minus the difference in the simulated “control” (C) hotspots within realms. The difference in the “control” hotspots was calculated for each biogeographic stochastic mapping by subtracting the estimated age of colonization of the non-hotspot region from the estimated age of colonization of the hotspot region (n = 50 replicates × 50 control regions). Solid line shows the median, box limits indicate the first and third quartiles.

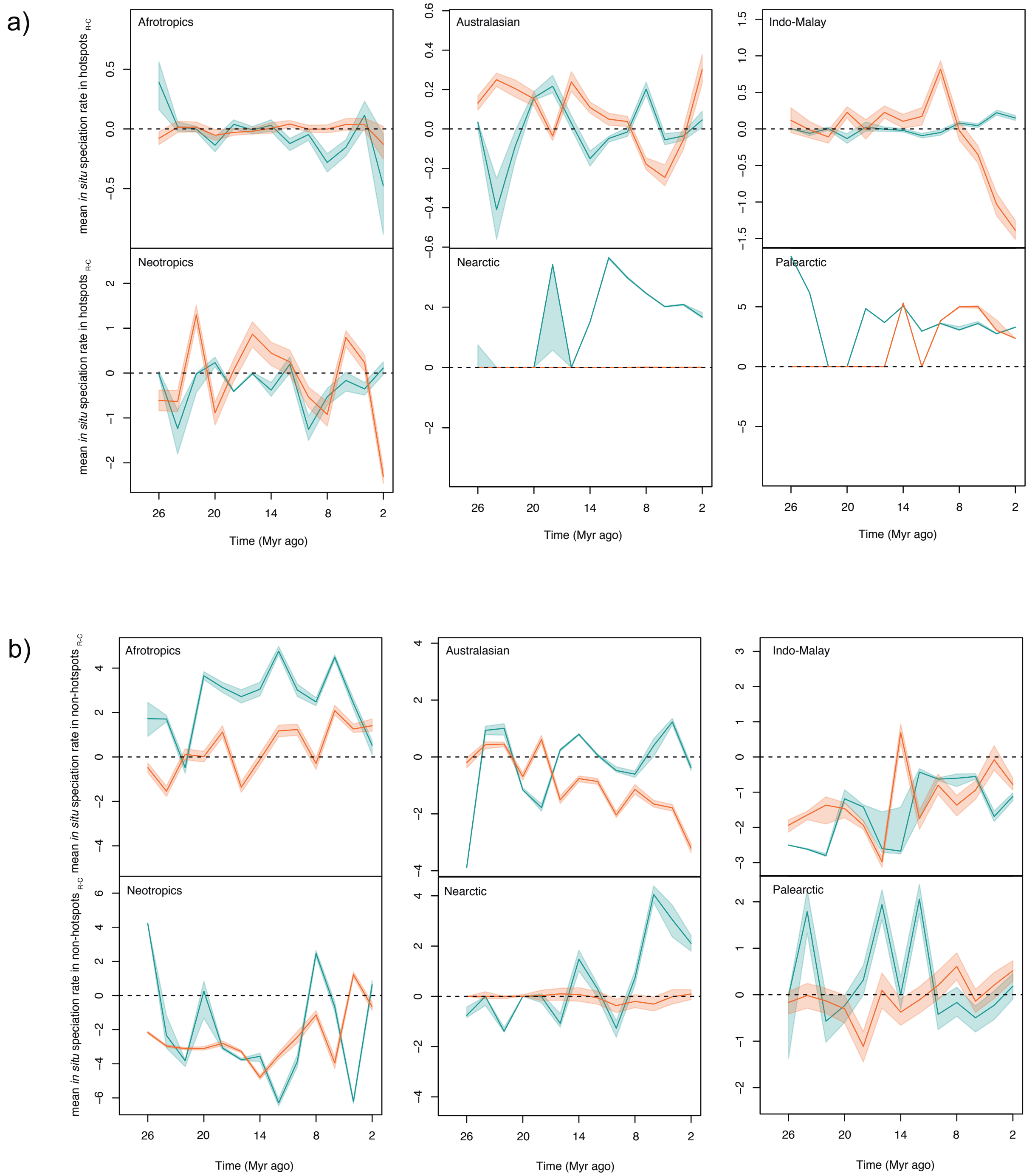


**Fig. S4. Empirically estimated in situ cladogenetic rates in hotspots and non-hotspots differ from rates estimated in "control areas" with similar size and spatial structure to the real**

**hotspots.** Differences for *in situ* cladogenetic rates in the hotspots (**a**) and in the non-hotspots (**b**) calculated with the real (R) dataset minus the simulated "control" (C) hotspots within each realm. Solid lines indicate the median difference in the *in situ* rates at a particular time bin and the shaded areas indicate the 90% confidence interval. Intervals overlapping the dotted line indicate a lack of statistically significant differences at $\alpha = 0.10$. The differences have been standardized by dividing the values by the realm-specific standard deviations.

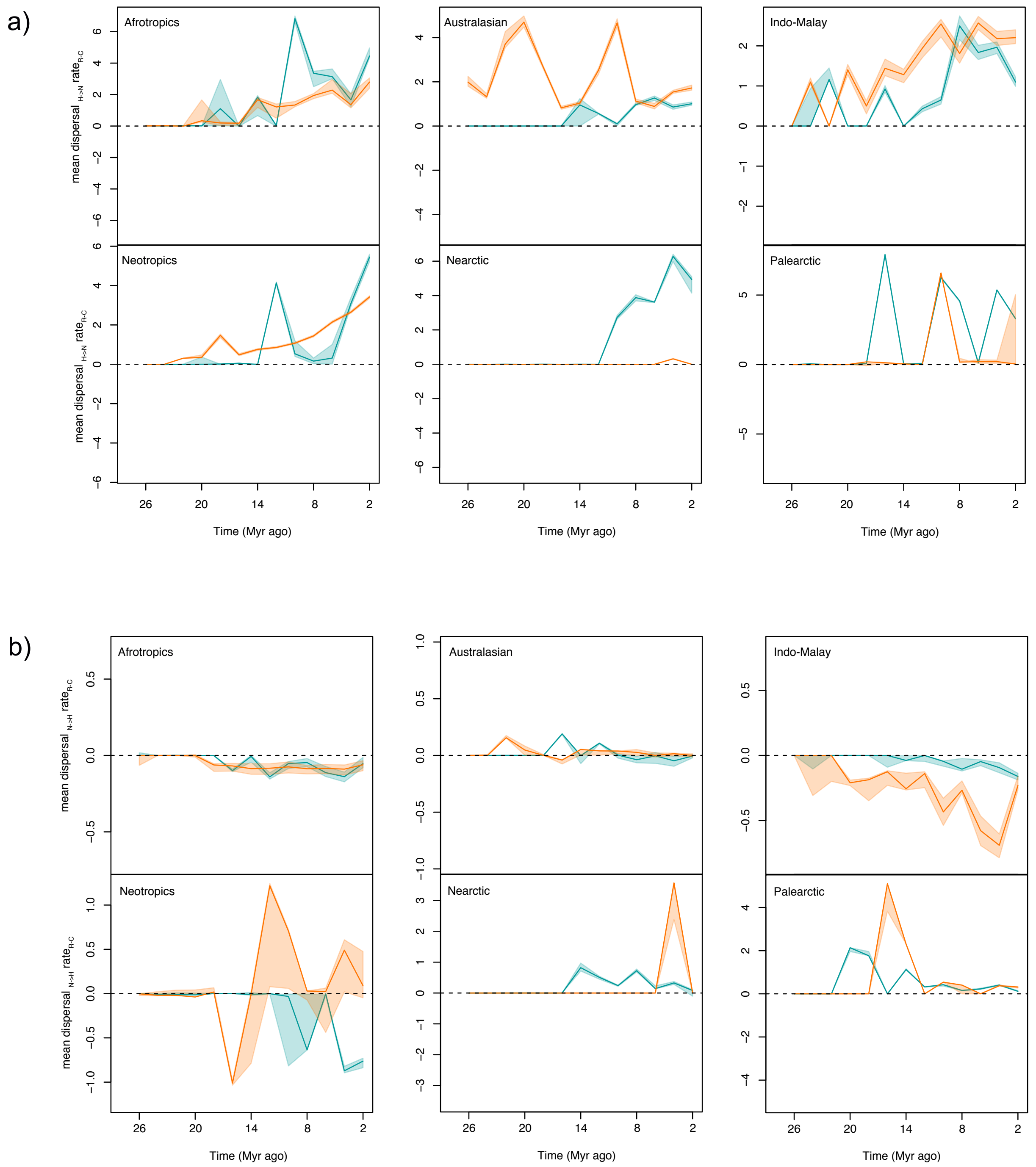


**Fig. S5. Empirically estimated dispersal rates from hotspots to non-hotspots (H → N) and from non-hotspots to hotspots (N → H) differ from rates estimated in control areas with similar size and spatial structure to the real hotspots.** Differences for dispersal rates from

hotspots to non-hotspots (H→N) (**a**) and from non-hotspots to hotspots (N→H) (**b**) calculated with the real (R) dataset minus the simulated "control" (C) hotspots within realms. Lines and shaded areas are presented as in fig. S4.

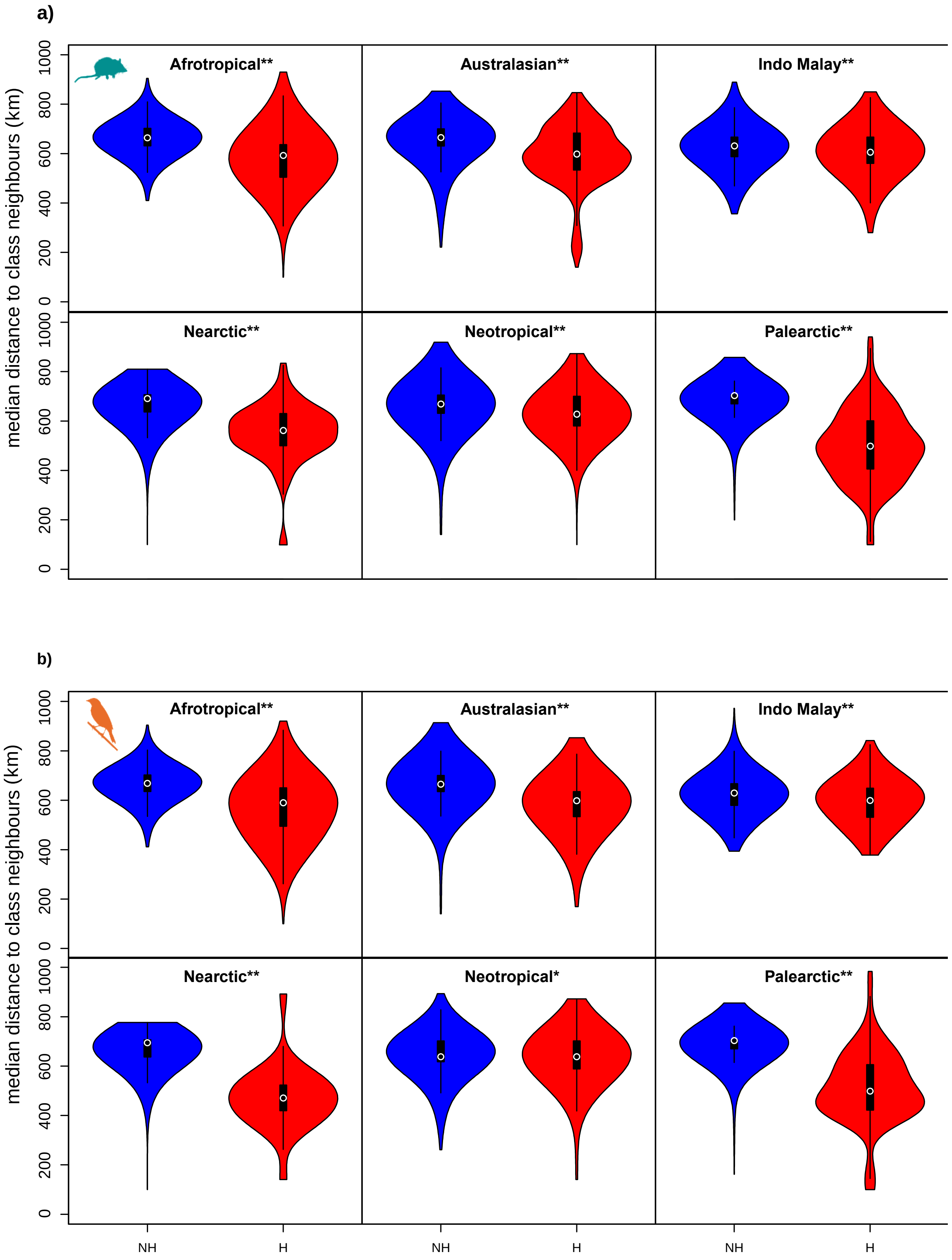
a)
b)
median distance to class neighbours (km)
0
200
400
600
800
1000
Afrotropical**
Australasian**
Indo Malay**
Nearctic**
Neotropical**
Palearctic**
Neotropical*
NH
H

**Fig. S6. Similar differences in contiguity of hotspot and non-hotspot cells across biogeographic realms.** For each realm, the median distance of each cell to every neighboring cell of the same class in a 1000 km radius is shown for hotspots (red) and non-hotspots (blue) for **a**) mammals, and **b**) birds. Asterisks indicate significant differences (* = p-value < 0.05; ** = p-value <0.01) between hotspot and non-hotspot contiguity with a *t*-test.

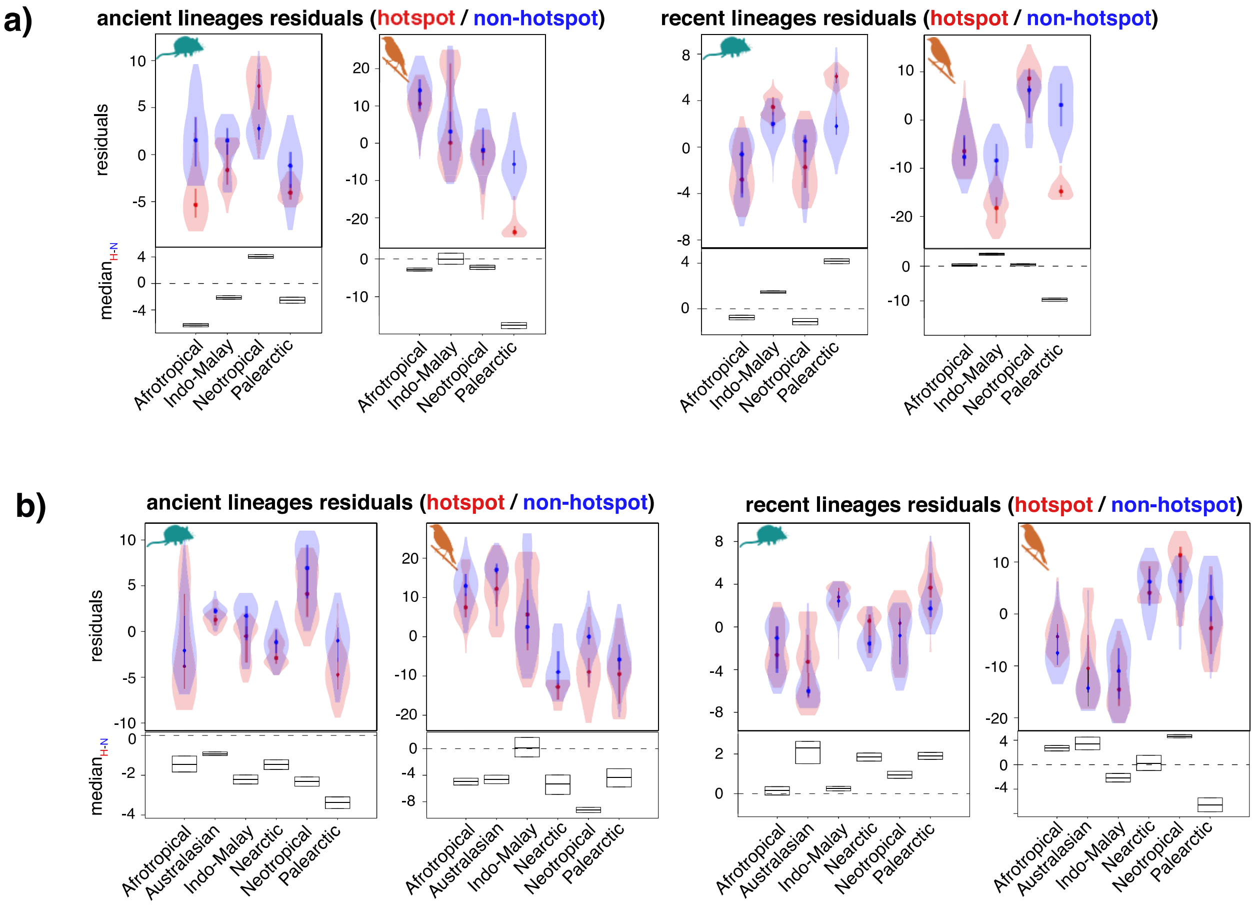


**Fig. S7. Species richness-based hotspots and narrow ranged species-based hotspots are poor in ancient lineages and sometimes rich in recent lineages.** Residuals from linear models predicting cell-specific richness of ancient and recent lineages in hotspots (H, shown in red) and non-hotspot regions (N, shown in blue) for species richness-based (a) and narrow ranged species-based (b) hotspots. Positive residuals indicate a regional excess of ancient/recent lineages and negative residuals indicate a deficit. Lower panels are presented as in Fig. 1.

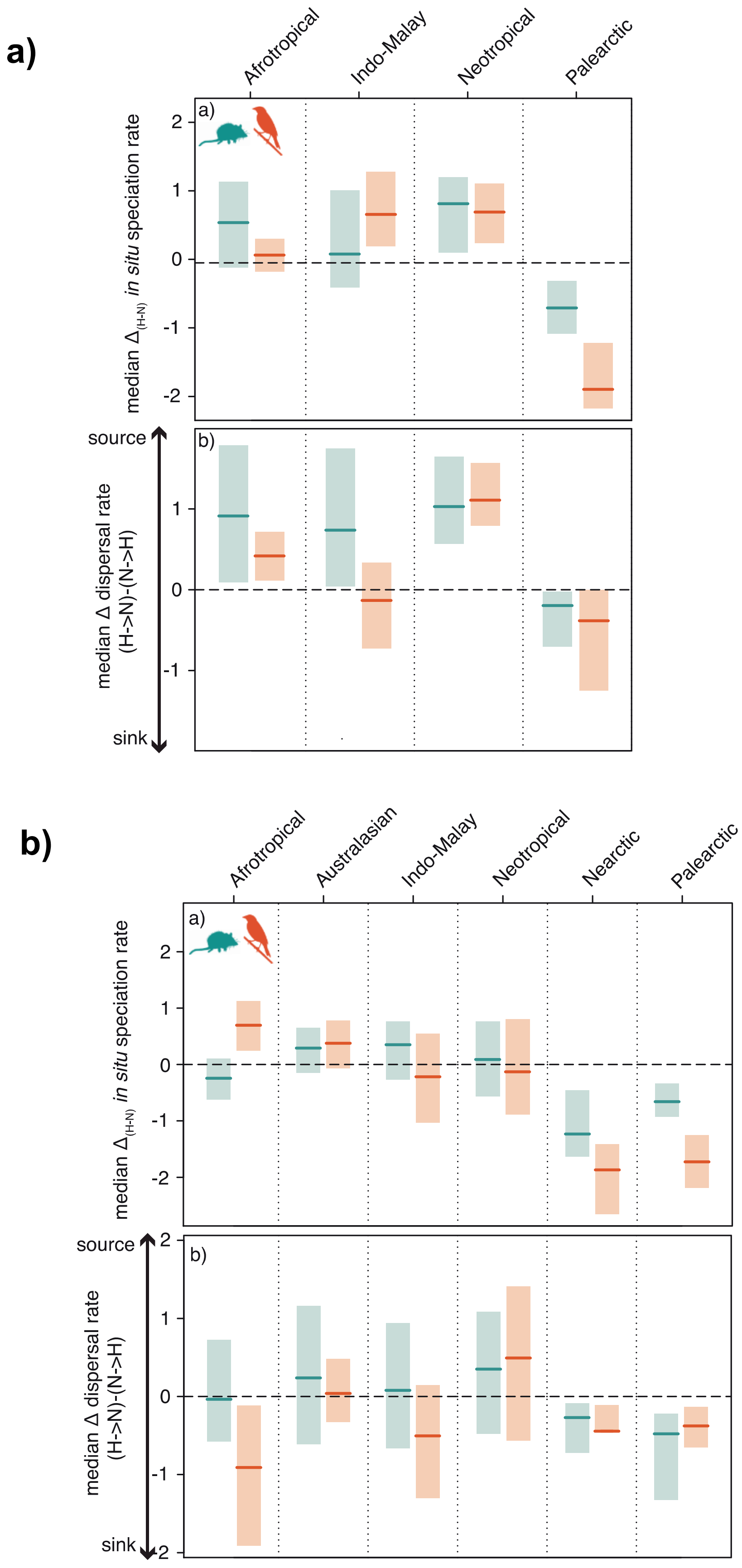

a)
Afrotropical
Indo-Malay
Neotropical
Palearctic
a)
median $\Delta_{(H\text{-}N)}$ *in situ* speciation rate
2
1
0
-1
-2
b)
source
sink
median Δ dispersal rate (H->N)-(N->H)
1
0
-1
b)
Afrotropical
Australasian
Indo-Malay
Neotropical
Nearctic
Palearctic
a)
median $\Delta_{(H\text{-}N)}$ *in situ* speciation rate
2
1
0
-1
-2
b)
source
sink
median Δ dispersal rate (H->N)-(N->H)
2
1
0
-1
-2

**Fig. S8. Contrasting macroevolutionary routes in species richness-based hotspots and non-hotspots and in narrow ranged species-based hotspots and non-hotspots.** *In situ* cladogenesis rates between 2 to 26 Ma ago within non-hotspots were subtracted from rates within hotspots in each of six biogeographic realms (top panels), and divided by the overall standard deviation to allow for comparison across realms. Species richness-based and narrow ranged species-based hotspots shown in (a) and (b), respectively. Dispersal rates between 2 to 26 Ma ago from non-hotspots to hotspots (N→H) were subtracted from hotspot to non-hotspots (H→N) rates within each realm (bottom panels).  Lines and shades are presented as in Fig. 2

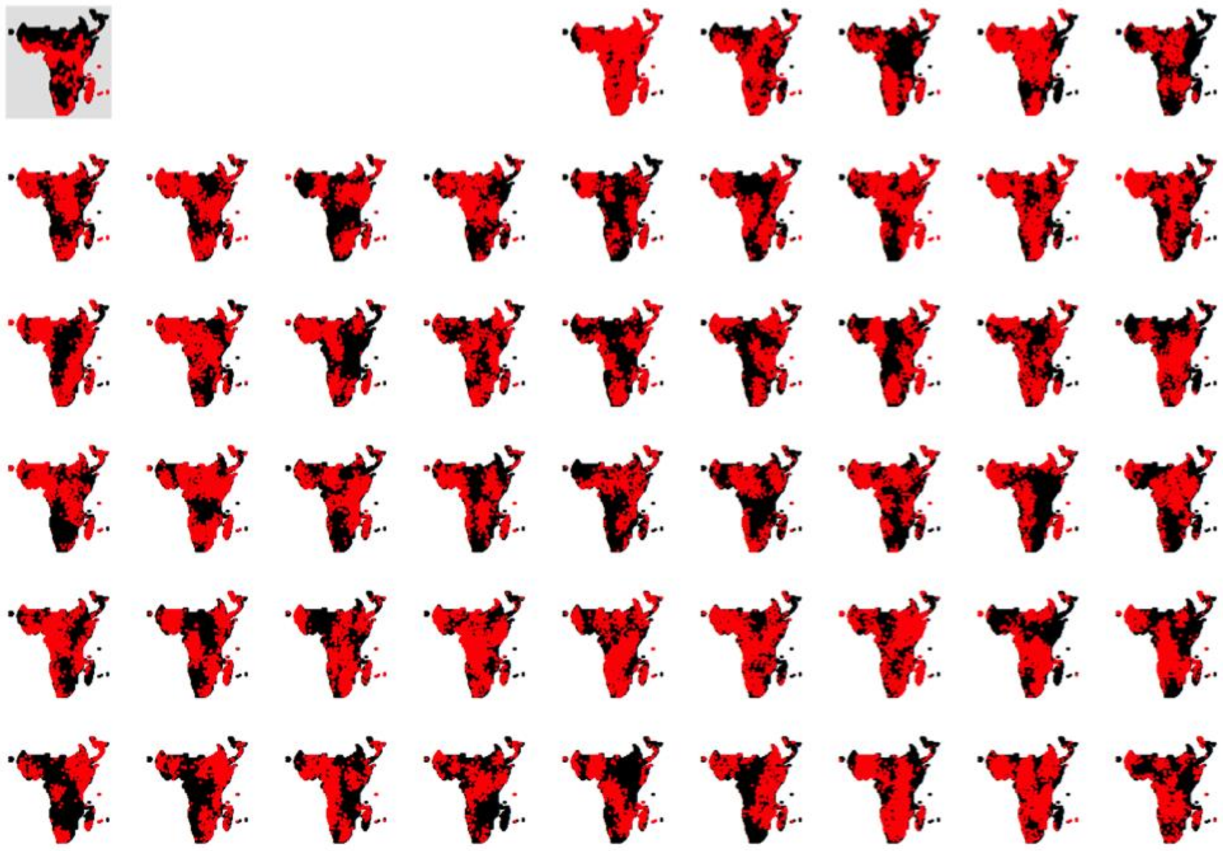

**Fig. S9. Example of simulating control hotspots.** Map of hotspots (shown in red) in the Afrotropics (observations outlined in grey) and 50 sets of cells with similar size and spatial structure shown alongside.

**Table S1. DR and BAMM produce consistent differences between hotspot and non-hotspot regions.** For each region, we subtracted the non-hotspot residuals from hotspots and compared the sign between the DR and BAMM approaches. A match indicates that the sign of the comparison is the same in DR and BAMM (e.g., there are less ancient lineages in the hotspots both with DR and BAMM estimates). A mismatch indicates that the sign of the comparison is not the same (e.g., there are less ancient lineages in the hotspots if estimated with DR but more ancient lineages if estimated with BAMM).

| | MAMMALS | | BIRDS | |
|---|---|---|---|---|
| | Ancient lineages residuals | Recent lineages residuals | Ancient lineages residuals | Recent lineages residuals |
| MATCH | 4 | 4 | 2 | 4 |
| NO MATCH | 2 | 1* | 2* | 1* |

* indicates nonsensical comparison(s) (i.e., when one of the values in a comparison overlaps zero, so we cannot tell if it is larger or smaller in the hotspots)

**Table S2. Total size and proportion of hotspot cells across biogeographic realms.**

| Realm name | Size (# of cells) | Proportion of hotspots |
|---|---|---|
| Afrotropical | 2512 | 0.29 |
| Australasian | 1337 | 0.32 |
| Indo-Malay | 1255 | 0.50 |
| Nearctic | 2753 | 0.08 |
| Neotropical | 2304 | 0.46 |
| Palearctic | 6277 | 0.03 |

**Table S3. Mean of median distances (kilometer) of each cell to every neighboring cell of the same class with a radius of 1000 km is shown for hotspots and non-hotspots for mammals and birds.**

| | mammals | | birds | |
|---|---|---|---|---|
| | Distance to non-hotspot | Distance to hotspot | Distance to non-hotspot | Distance to hotspot |
| Afrotropical | 661.34 | 584.04 | 666.37 | 567.99 |
| Australasian | 651.95 | 591.99 | 666.74 | 581.84 |
| Indo-Malay | 623.76 | 605.12 | 621.29 | 596.61 |
| Nearctic | 663.52 | 550.97 | 665.32 | 470.09 |
| Neotropical | 661.37 | 628.61 | 643.00 | 634.51 |
| Palearctic | 685.80 | 496.45 | 686.73 | 509.50 |

**Table S4. Overlap of WE-based hotspots with SR- and NRS-based hotspots.** Size is the number of grid cells.

| | mammals | | | | | birds | | | | |
|---|---|---|---|---|---|---|---|---|---|---|
| | WE hotspots (size) | SR hotspots (size) | overlap (proportion WE ∩ SR) | NR hotspots (size) | overlap (proportion WE ∩ NR) | WE hotspots (size) | SR hotspots (size) | overlap (proportion WE ∩ SR) | NR hotspots (size) | overlap (proportion WE ∩ NR) |
| Afrotropics | 735 | 1129 | 0.73 | 549 | 0.64 | 651 | 1162 | 0.76 | 356 | 0.44 |
| Australasian | 433 | 23 | 0.05 | 391 | 0.77 | 555 | 113 | 0.20 | 428 | 0.72 |
| Indo-Malay | 629 | 472 | 0.68 | 390 | 0.58 | 623 | 433 | 0.65 | 351 | 0.48 |
| Nearctic | 186 | 23 | 0.00 | 177 | 0.64 | 27 | 0 | 0.00 | 46 | 0.48 |
| Neotropical | 1064 | 1531 | 0.75 | 844 | 0.68 | 1298 | 1504 | 0.76 | 706 | 0.49 |
| Palearctic | 206 | 92 | 0.30 | 358 | 0.73 | 98 | 75 | 0.61 | 92 | 0.38 |

**Table S5. Model fit of BioGeoBEARS in six biogeographic realms.** Best fitting models according to AICc are shaded.

| | **Afrotropics** | | **Australasian** | | **Indo-Malay** | | **Nearctic** | | **Neotropics** | | **Palearctic** | |
|---|---|---|---|---|---|---|---|---|---|---|---|---|
| model | mammals | birds | mammals | birds | mammals | birds | mammals | birds | mammals | birds | mammals | birds |
| DEC | 14694 | 33464 | 12342 | 28771 | 12495 | 28997 | 12270 | 26217 | 14985 | 34529 | 12130 | 26797 |
| DEC+J | 13620 | 31236 | 11073 | 25744 | 11032 | 25860 | 10876 | 23127 | 14041 | 32325 | 10711 | 23727 |
| DIVALIKE | 15300 | 34723 | 12925 | 30040 | 12952 | 29984 | 12815 | 27430 | 15645 | 35945 | 12649 | 27990 |
| DIVALIKE+J | 13958 | 31994 | 11346 | 26362 | 11218 | 26312 | 11128 | 23630 | 14463 | 33214 | 10925 | 24237 |
| BAYAREALIKE | 15399 | 34012 | 13744 | 31789 | 14538 | 32939 | 13954 | 29596 | 14894 | 36114 | 14003 | 30516 |
| BAYAREALIKE+J | 12751 | 27798 | 11256 | 25874 | 11237 | 25958 | 11013 | 23217 | 12737 | 31418 | 11004 | 23895 |
